# Performance evaluation of high-volume electret filter air samplers in aerosol microbiome research

**DOI:** 10.1186/s40793-020-00362-x

**Published:** 2020-07-28

**Authors:** Kari Oline Bøifot, Jostein Gohli, Gunnar Skogan, Marius Dybwad

**Affiliations:** 1 Norwegian Defence Research Establishment FFI, P O Box 25, NO-2027 Kjeller, Norway; 2 Department of Analytics, Environmental & Forensic Sciences, King’s College London, 150 Stamford Street, London, SE1 9NH UK

**Keywords:** Air, Bioaerosol, ACD-200 bobcat, DNA, Electret, Filter, High-volume, Microbiome, Sampler, SASS 3100, Shotgun metagenomic sequencing

## Abstract

**Background:**

Reliable identification and quantification of bioaerosols is fundamental in aerosol microbiome research, highlighting the importance of using sampling equipment with well-defined performance characteristics. Following advances in sequencing technology, shotgun metagenomic sequencing (SMS) of environmental samples is now possible. However, SMS of air samples is challenging due to low biomass, but with the use of high-volume air samplers sufficient DNA yields can be obtained. Here we investigate the sampling performance and comparability of two hand-portable, battery-operated, high-volume electret filter air samplers, SASS 3100 and ACD-200 Bobcat, previously used in SMS-based aerosol microbiome research.

**Results:**

SASS and Bobcat consistently delivered end-to-end sampling efficiencies > 80% during the aerosol chamber evaluation, demonstrating both as effective high-volume air samplers capable of retaining quantitative associations. Filter recovery efficiencies were investigated with manual and sampler-specific semi-automated extraction procedures. Bobcat semi-automated extraction showed reduced efficiency compared to manual extraction. Bobcat tended towards higher sampling efficiencies compared to SASS when combined with manual extraction. To evaluate real-world sampling performance, side-by-side SASS and Bobcat sampling was done in a semi-suburban outdoor environment and subway stations. SMS-based microbiome profiles revealed that highly abundant bacterial species had similar representation across samplers. While alpha diversity did not vary for the two samplers, beta diversity analyses showed significant within-pair variation in subway samples. Certain species were found to be captured only by one of the two samplers, particularly in subway samples.

**Conclusions:**

SASS and Bobcat were both found capable of collecting sufficient aerosol biomass amounts for SMS, even at sampling times down to 30 min. Bobcat semi-automated filter extraction was shown to be less effective than manual filter extraction. For the most abundant species the samplers were comparable, but systematic sampler-specific differences were observed at species level. This suggests that studies conducted with these highly similar air samplers can be compared in a meaningful way, but it would not be recommended to combine samples from the two samplers in joint analyses. The outcome of this work contributes to improved selection of sampling equipment for use in SMS-based aerosol microbiome research and highlights the importance of acknowledging bias introduced by sampling equipment and sample recovery procedures.

## Background

The ability to reliably identify and quantify biological aerosols (bioaerosols) is a fundamental enabler in aerosol microbiome research. This highlights the importance of using air samplers with well-defined performance characteristics, regardless of downstream analysis techniques, since this is the only way to ensure capture of representative samples that retain a reliable quantitative association between the collected sample and the sampled environment [1–4].

Bioaerosol research has traditionally relied on culturing for quantification and identification of airborne microorganisms, and while culture techniques are still in use, the rise of the molecular era has introduced several culture-independent molecular techniques that have revolutionized the field of microbiology [5]. Molecular techniques offer several advantages, e.g. reduced costs, increased speed, and improved data quality and quantity, and include powerful DNA-based methods such as real-time quantitative polymerase chain reaction (qPCR) and high-throughput sequencing (HTS) [6].

Although airborne microorganisms are ubiquitous in almost any indoor and outdoor environment, air is recognized as a low biomass environment relative to soil, feces and water [7]. The ability to recover sufficient biomass from air in a state suitable for downstream analyses has therefore been a recurring challenge in bioaerosol research [8]. In the context of traditional culture-based techniques, the main challenge has generally been to avoid altering the biological state of the collected biomass, e.g. reduced viability/culturability due to desiccation and sampling stress, since this would bias the culture results. However, in the context of culture-independent DNA-based methods, which are mostly insensitive to the exact biological state of the collected biomass, the main challenge has been to collect sufficient biomass to facilitate reliable downstream analyses.

Recent advances in the field of HTS have opened the door to direct shotgun metagenomic sequencing (SMS) of DNA isolated from complex environmental samples [9–12]. However, the required quality and quantity of input material for SMS is generally higher than for other DNA-based techniques such as qPCR and HTS-based amplicon sequencing. Thus, few bioaerosol investigations have succeeded in adopting direct SMS to study aerosol microbiomes [13–16]. Several strategies including long-duration air sampling (days to weeks), sample pooling, cloning, and whole genome amplification (WGA) techniques have been used to mitigate the low biomass challenge [13, 14, 17, 18]. Each strategy is however associated with inherent drawbacks, e.g., long-duration sampling may increase the contamination risk and compromise the integrity and stability of the collected material, long-duration sampling and sample pooling typically come at the expense of spatiotemporal resolution, and WGA techniques may increase the risk of amplification-related bias [1, 19]. A different strategy to increase DNA yields involves efforts to optimize the post-sampling processing steps (e.g. DNA extraction) and to maximize biomass collection [15, 16, 20]. Air samplers with high flowrates and high sampling efficiencies are favorable when the goal is to maximize the rate of biomass collection from air [2].

Air sampling equipment comes in several varieties and rely on a wide range of collection principles including impaction, impingement, centrifugation, filtration and electrostatic precipitation [1]. Filtration and liquid impingement are two sampling principles commonly used in combination with molecular analyses [1, 21]. An inherent drawback with traditional filter-based air samplers has been flowrate limitations due to the use of filter materials with low porosity resulting in a high pressure-drop, and the need for long-duration sampling [1]. For liquid impingers and wetted-wall cyclones, collection of biomass has usually been limited by short sampling times due to evaporation of sampling medium. One strategy to overcome this issue has been to replenish the sampling liquid with either water or sampling medium/buffer [1, 4]. However, this complicates the fluidics system, may increase the contamination risk, and could concentrate buffer salts and impurities. Another concern with evaporation of sampling medium is reaerosolization of collected particles [4, 22]. A recent study has shown that non-random reaerosolization of certain taxonomic groups in liquid samplers can introduce substantial bias when investigating microbial diversity [23]. Also, both Lemieux et al. [23] and Mbareche et al. [3] have reported a significantly higher microbial diversity with the use of an electret filter sampler compared to a wet cyclone sampler when sampling side-by-side in real-world environments.

The electret filter technology has recently received increased attention [3, 23–28], and has also been used in aerosol microbiome studies utilizing direct SMS [14, 16]. The technology relies on low pressure-drop microfibrous filters where each fiber has an electric field frozen into it. This induces a charge in aerosol particles passing through the filter, resulting in an electret capture mechanism. Air samplers based on electret filters provide a combination of equipment and sampling properties well suited for applications demanding a flexible and mobile platform, due to the high flowrate, small size, low weight, low power consumption, high sampling efficiency, and flexible sampling time including the possibility of long-duration continuous sampling [2, 3, 26]. The high flowrates and flexible sampling times support collection of high biomass yields, which are particularly important when combining low biomass air environments with analyses requiring high biomass inputs such as SMS.

The aim of this study was to characterize and compare the sampling performance of SASS 3100 and ACD-200 Bobcat, both high-volume electret filter air samplers, recently used in the context of SMS-based aerosol microbiome research. To establish accurate physical sampling efficiencies (the combination of collection and recovery efficiencies), as a proxy for total biomass sampling efficiency, the air samplers were benchmarked against gelatin reference filters in a controlled aerosol chamber using 1 and 3 μm particles containing fluorescent and bacterial spore tracers. Additionally, air samples were collected side-by-side with SASS and Bobcat in an outdoor semi-suburban environment and in subway stations with expected low biomass. Total DNA, bacterial 16S rRNA gene copy yields and SMS-based aerosol microbiome profiles were compared across paired air samples.

## Methods

### Evaluated air samplers

**Fig. 1:**
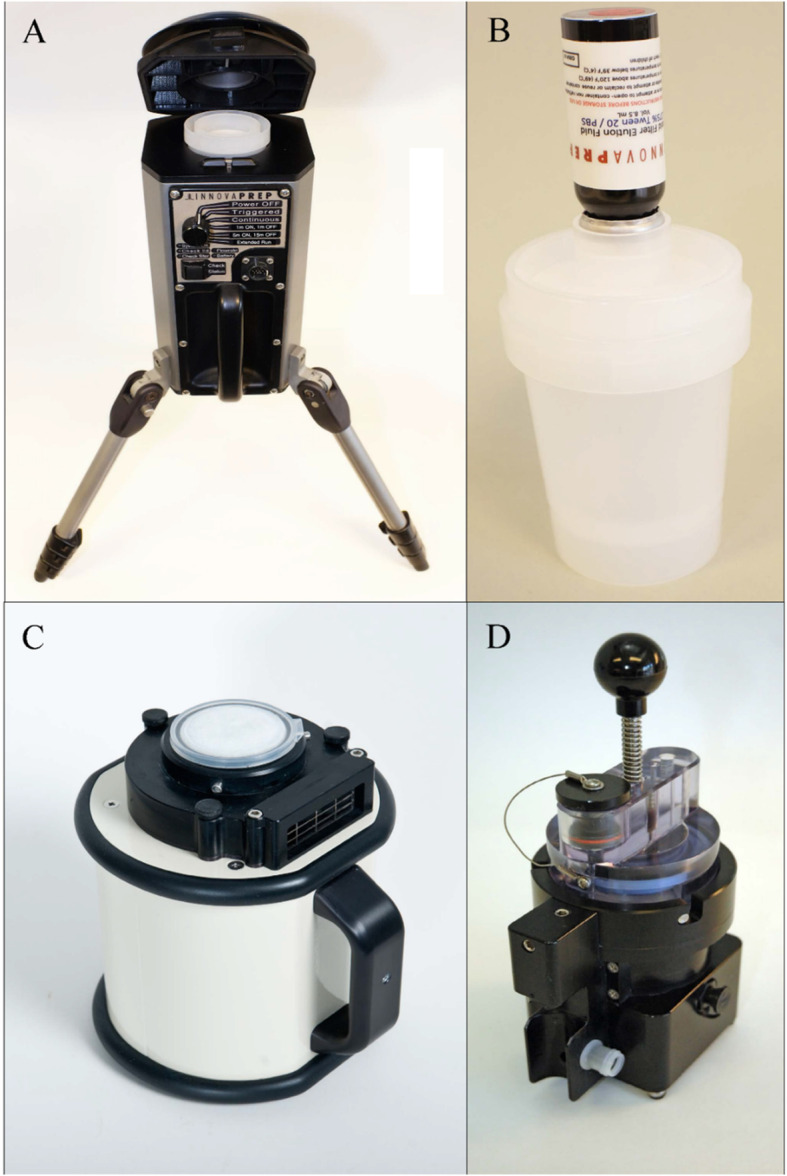
Evaluated high-volume electret filter air samplers. The ACD-200 Bobcat (**a**) and SASS 3100 (**c**) high-volume electret filter air samplers evaluated in this study. For semi-automated filter extraction, Bobcat filters can be used in combination with Bobcat Rapid Filter Elution kits (**b**), while SASS filters can be used in combination with the SASS 3010 Particle Extractor (**d**)

Two commercial high-volume electret filter air samplers were evaluated, namely SASS 3100 from Research International, Monroe, WA, USA (Fig. 1, Panel c) and ACD-200 Bobcat from Innovaprep, Drexel, MO, USA (Fig. 1, Panel a) [29, 30]. SASS can be operated at a user-adjustable flowrate of 50–300 l of air per minute (LPM), while Bobcat can be operated at 200 LPM in continuous sampling mode. To achieve the highest possible biomass collection rate, the maximum flowrate was used for SASS (300 LPM) in this study. Both samplers come with support for sampler-specific semi-automated filter extraction (filter-to-liquid) to generate liquid samples for downstream analysis. SASS can be used in combination with a SASS 3010 Particle Extractor and Extraction kits containing electret filter and extraction consumables (Fig. 1, Panel d), while Bobcat can be used in combination with Rapid Filter Elution kits containing electret filter and extraction consumables (Fig. 1, Panel b). In this study, the SASS 3100 was used in combination with electret filters from Research International (P/N: 7100–134–232-01), while the ACD-200 Bobcat was used in combination with electret filters from Innovaprep (P/N: AC00201-P).

### Aerosol chamber-based sampling efficiency evaluation

SASS and Bobcat were subjected to aerosol chamber-based test and evaluation to establish physical sampling efficiencies. Fluorescent (Uranine) and aerostable biological (BG spores) tracers were used to determine physical sampling efficiencies for 1 μm and 3 μm aerosol particles relative to gelatin reference filters and expressed as percent (%) relative sampling efficiency. The gelatin reference filters were benchmarked against two other commonly-used reference samplers, SKC BioSampler and isopore membrane filters, and were shown to have a consistent sampling efficiency close to 100% (Supplementary Text 1). Sampling efficiencies for SASS and Bobcat were determined with both a common manual filter extraction procedure and sampler-specific semi-automated filter extraction procedures to investigate potential differences in recovery efficiency. For each of the four test conditions (1 μm and 3 μm semi-automated, and 1 μm and 3 μm manual extraction) 15 SASS filters and 13 Bobcat filters were analyzed for Uranine and BG spores. Since the SASS has a user-adjustable sampling flow rate, the sampling efficiency at different sampling flowrates (50, 100, 200 and 300 LPM) was also investigated (Supplementary Text 2).

#### Test materials and spray liquid formulation

Bacterial spores (*Bacillus atrophaeus*, formerly *Bacillus globigii*, BG) were used as biological test material. A freeze-dried powder containing 2.0 × 10^8^ colony forming units per milligram (cfu mg^− 1^) of BG spores (DPG Lot 19,076–03268) was provided by Dugway Proving Ground (DPG, Dugway, UT, USA). A stock solution of BG spores (5 mg ml^− 1^) was prepared by suspending in MilliQ water (Millipore, Billerica, MA, USA) assisted by vortexing (1 min). BG spores were washed by centrifugation (3000×g, 10 min), resuspended in MilliQ water and stored at 4 °C. Uranine (Fluorescein sodium salt) was used as fluorescent test material. A 5 mg ml^− 1^ stock solution of Uranine AP (C.I. 45,350, Millipore) in MilliQ water was prepared and stored at 4 °C. Spray solutions were prepared fresh each day by diluting stock solutions of BG spores/Uranine AP in MilliQ water to concentrations of 0.15/0.1 mg ml^− 1^ (~ 1 μm particles) and 1.4/0.4 mg ml^− 1^ (~ 3 μm particles).

#### Aerosol test facility

Air sampler testing was done in a 12 m^3^ (3 × 2 × 2 m) stainless steel aerosol test chamber (ATC, Dycor Technologies, Edmonton, AB, Canada) fitted with external heating, ventilation and air conditioning (HVAC) and high-efficiency particulate air (HEPA)-filtration systems. The ATC was equipped with two mixing fans (120 mm), meteorology sensors for temperature, humidity and pressure, optical particle counter (Grimm 1.108, Grimm Technologies, Douglasville, GA, USA), aerodynamic particle sizer (APS 3321, TSI, Shoreview, MN, USA), and two slit-to-agar samplers (STA-203, New Brunswick, Edison, NJ, USA). Real-time monitoring of test aerosol concentration and particle size distribution was done with Grimm 1.108 and APS 3321.

#### Aerosol generation

The targeted aerosol particle sizes of 1 μm and 3 μm count median aerodynamic diameter (CMAD) was produced with 120- and 48-kHz ultrasonic atomizer nozzles (Sono-Tek, Milton, NY, USA), respectively. The ultrasonic atomizers were powered by an ECHO multiband ultrasonic generator (Model 06–05-00330, Sono-Tek). Spray solution was loaded into 50 ml luer lock syringes and the ultrasonic atomizer was fed with a syringe feeder (Model 997E, Sono-Tek). Appropriate instrument settings for the ATC and its subsystems were determined during pre-study experiments to generate reproducible aerosol concentration levels and size distributions, and then kept static throughout the study. Dissemination with 120-kHz ultrasonic atomizer (1 μm particles) was performed for 2 min at 500 μl min^− 1^ with ultrasonic generator set to 3 W. Dissemination with 48-kHz ultrasonic atomizer (3 μm particles) was performed for 30 s at 400 μl min^− 1^ with ultrasonic generator set to 4 W. Throughout the experiments the ATC was continously stirred using the internal mixing fans to produce stirred settling sampling conditions. After dissemination, the ATC was homogenized for 1 min before initiating sampling. The air flow inside the ATC was measured with a VTS KS 200 hot-wire anemometer (KIMO Canada, Québec, Canada) at different heights and positions in the visinity of the sampling equipment and shown to be < 0.7 m s^− 1^. Particle size distributions were calculated based on APS 3321 measurements from at least five experiments and reported as mean (± standard deviation) CMAD (μm) and geometric standard deviation (unitless; Table S1).

#### Aerosol collection

SASS, Bobcat and gelatin reference filters (Sartorius, Germany) were positioned with equal inlet heights (30–40 cm above floor level) and at alternating sampling positions inside the ATC. SASS and Bobcat were powered by UBI-2590 lithium-ion rechargeable batteries (Ultralife batteries, NY, USA) and operated at flowrates of 300 and 200 LPM, respectively. Gelatin reference filters were mounted on a porous plastic support pad in a 2-piece conductive filter cassette (SKC 225–8496, SKC Inc., PA, USA) operated by a vacuum pump (Gast Manufacturing, MI, USA). The flowrate was adjusted to 10 LPM with a Sierra Top-Trak 820 Series thermal mass flow meter (Sierra Instruments, CA, USA). Each aerosol trial consisted of 5 min simultaneous collection with all air samplers. The ATC was purged after each trial before samples were recovered and the samplers reloaded.

#### Manual filter extraction

Collected particles were extracted from SASS and Bobcat filters with a common manual filter extraction procedure. Liquid extraction was done by removing the filters from their housing and transferring them into 50 ml polypropylene vials pre-loaded with 10 ml PBSTA, PBS with 0.05% Triton X-100 (Sigma-Aldrich, St. Louis, MO, USA) and 0.005% Antifoam A (Sigma-Aldrich). The vial was shaken by hand to wet the filter and then vortexed (Reax Top, Heidolph Instruments, Schwabach, Germany) at maximum speed for 20 s. Sterile forceps was use to transfer the filter into a 10 ml syringe to extract residual liquid in the filter back into the vial before discarding the filter. The gelatin reference filter was transferred to a 50 ml polypropylene vial pre-loaded with 20 ml PBSTA and dissolved by incubating in water bath at 37 °C for 15 min. The manual extraction protocols have been optimized to produce maximized generic biomass recovery from electret and gelatin filters with demonstrated recovery efficiencies close to 100% (*data not shown*).

#### Semi-automated filter extraction

Collected particles were extracted from SASS and Bobcat filters in accordance with their respective semi-automated filter extraction procedures. SASS extraction was done in accordance with the manufacturer-recommended protocol using 8.5 ml PBSTA and SASS 3010 Particle Extractor (Fig. 1, Panel d) [30]. Bobcat extraction was done in accordance with the manufacturer-recommended protocol using Bobcat Rapid Filter Elution kits (Fig. 1, Panel b) [29].

#### Plate count analysis

BG spore concentrations in filter extracts were assayed using standard plate counting and expressed as cfu L^−1^of sampled air. Serial dilutions were plated in triplicates on trypticase soy agar (TSA, BD Difco 236,950, Fisher Scientific, Pittsburgh, PA, USA) and incubated at 30 °C for 18 h before colony counting with a ProtoCol HR automated colony counter (Synbiosis, Cambridge, UK).

#### Fluorimeter analysis

Uranine concentrations in filter extracts were assayed using FLUOStar Optima microplate fluorimeter (BMG Labtech, Offenberg, Germany). An aliquot (200 μl) of each filter extract was diluted with PBSTA and mixed 1:2 with 0.1 M Tris-base buffer, pH 9.5 (Sigma-Aldrich). Each sample was measured in triplicates (100 μl) using Corning 3915 black 96-well microplates (Sigma-Aldrich). To generate a standard curve, Uranine was serially diluted in PBSTA and mixed 1:2 with 0.1 M Tris-base buffer, pH 9.5, and analyzed in the same way as the filter extracts. Uranine concentrations were expressed as mg L^− 1^ of sampled air.

#### Statistical analyses

The aerosol chamber data, i.e., the sampling efficiencies for SASS and Bobcat, were analyzed with nonparametric two-sample Kolmogorov-Smirnov tests in R, which compares empirical cumulative distributions to test for differences between two groups.

### Evaluation of sampling performance in outdoor semi-suburban and subway air

To evaluate the performance of SASS and Bobcat in real-world sampling conditions, i.e., complex low biomass environments, the samplers were operated side-by-side. Twelve paired air samples were collected with SASS and Bobcat side-by-side for 6–8 h on each occasion, in an outdoor semi-suburban environment (Kjeller, Norway, 59.976540 N, 11.048691E) from late January to late May 2017 (Table S2). Additionally, paired air samples from eight different subway stations in Oslo, Norway were collected on June 21, 2017 with a 30-min sampling time (Table S3). Sampling performance was assessed by comparing bacterial 16S rRNA gene copy yields per cubic meter of sampled air (only for outdoor samples), total DNA per cubic meter of air and by SMS-based aerosol microbiome profiling.

#### Aerosol collection

The samplers were mounted on tripods with an inlet height of ~ 1.5 m. Since SASS was not fitted with a dust/rain screen, it was operated with the inlet facing down (45 °C from down) to avoid filter deposition of large particles. Both samplers were otherwise operated as described for the aerosol chamber testing. The SASS inlet was wiped clean with 70% EtOH before filters were mounted onto the sampler. For the Bobcat there is very limited direct contact between sampler and single-use filter assembly. No between-sample cleaning is recommended in the Bobcat user guide and was therefore not performed. The filters were placed in 50 ml polypropylene vials and stored at − 80 °C until further processing. Negative controls (field blanks) were generated by opening and handling SASS and Bobcat filters at the sampling location.

#### DNA isolation subway air samples

Filter extraction and DNA isolation was performed as described by Bøifot et al. [16]. Briefly, liquid filter extraction was performed with NucliSENS lysis buffer (10 ml, BioMérieux, Marcy-l’Étoile, France) before the samples were centrifuged (30 min, 7000 x g). The filter extract supernatant was transferred to a fresh 50 ml tube, while the pellet was transferred to a microcentrifuge tube with PBS (1 ml, pH 7.5, Sigma-Aldrich, St. Louis, MO, USA), centrifuged (5 min, 17,000 x g) and supernatant combined with the filter extract supernatant. The pellet was resuspended in PBS (150 μl) and incubated (35 °C, 1 h) with MetaPolyzyme (10 μl, 5 mg/ml, Sigma-Aldrich) and sodium azide (5 μl, 0.1 M, Sigma-Aldrich). The pellet sample was transferred to a ZR BashingBead Lysis Tubes (0.1/0.5 mm beads, Zymo Research, Irvine, CA, USA) filled with PowerBead Solution (550 μl, Qiagen, Hilden, Germany) and Solution C1 (60 μl, Qiagen), and subjected to bead beating in a Mini-BeadBeater-8 (3 min, max intensity, BioSpec Products, Bartlesville, OK, USA). The bead tubes were centrifuged and the supernatant transferred before inhibitor removal with Solution C2 and C3 according to the DNeasy PowerSoil protocol (Qiagen). The purified lysate was pooled with the filter extract supernatant and DNA isolated according to the NucliSENS Magnetic Extraction Reagents kit (BioMérieux) with two modifications; 90 μl of silica beads were used, and the incubation with silica beads was increased to 20 min.

#### DNA isolation outdoor air samples

Liquid extraction of filter-collected particles was done as described for manual filter extraction, except for that NucliSENS lysis buffer (10 ml) was used instead of PBSTA. The liquid sample was centrifuged (6000 x g, 30 min) and the supernatant transferred to a fresh 50 ml polypropylene vial. The pellet was processed according to the lysis and inhibitor removal steps of the DNeasy PowerSoil kit. Briefly, the pellet was re-suspended in PowerBead Solution (550 μl) and transferred to autoclaved (121 °C, 45 min) bead tubes (2 ml, Sarstedt, Nümbrecht, Germany) filled with zirconia/silica beads (600 mg, 0.1 μm, BioSpec Products). Solution C1 (60 μl) was added and tubes subjected to bead beating (1 min, max intensity) in a Mini Bead Beater-8. The bead tubes were centrifuged (13,000 x g, 2 min) and the supernatant treated with Solution C2 and C3 according to the DNeasy PowerSoil protocol. The resulting lysate was pooled with the filter extract supernatant and DNA purified according to the manual protocol of the NucliSENS Magnetic Extraction Reagents kit.

#### Quantification of total DNA and bacterial 16S rRNA gene copies

DNA yield was determined with Qubit dsDNA HS assay (Life Technologies, Carlsbad, CA, USA) on Qubit 3.0 Fluorimeter (Life Technologies) and expressed as picograms per cubic meter of air. Bacterial 16S rRNA gene copy yield was determined (outdoor air samples only) with qPCR and expressed as 16S rRNA gene copies per cubic meter of air. The 16S rRNA gene qPCR assay was performed according to Liu et al. [31] on a LightCycler 480 (Roche Diagnostics, Oslo, Norway). Standard curve was generated with serial dilutions of *Escherichia coli* DNA (seven 16S rRNA gene copies per genome). Total DNA and 16S rRNA gene copies were compared between samplers using two-sample Kolmogorov-Smirnov tests.

#### SMS

DNA isolated from twelve paired outdoor air samples were subjected to SMS (150 bp paired-end) multiplexed on one lane (~ 300 M paired-end reads) on Illumina HiSeq 3000 (Illumina, San Diego, CA, USA). Library preparation was done with ThruPLEX DNA-Seq kit (Takara Bio, Mountain View, CA, USA) according to the recommended protocol and 20 amplification cycles. DNA samples from the subway environment were shipped to HudsonAlpha Genome Center (Huntsville, AL, USA) on dry ice for library preparation and shotgun sequencing as previously described [9, 32]. Demultiplexed raw sequence reads were quality trimmed (Q20) and adapters were removed with TrimGalore (v0.6.4) [33], a pearl wrapper for Cutadapt [34] and FastQC [35]. Reads mapping to PhiX (NCBI Accession: NC_001422) and the human genome (NCBI BioProject PRJNA31257) were removed from quality-filtered sequence reads (> 100 bp) using KneadData (v0.7.2) [36]. Taxonomic classification was performed with KrakenUniq [37] with a kmer length of 31 bp, using the NCBI RefSeq collection (protozoa, archaea, fungi, viral and bacterial; downloaded 2019-06-20) as a reference database. Filtering and merging of read statistics from individual sequence files were performed using a custom R script. Following Breitwieser et al. [37] we required taxa to have a number of unique kmers that exceeded 2000*sequence depth (M reads) in the input sequence files (filtering cutoffs where thus calculated on a per sample basis). For viruses, we observed this filtering to be too aggressive, and applied a separate filtering threshold of unique kmers > 30*sequence depth (M reads) for this kingdom. We also required that the number of mapped reads did no exceed 0.4 times the number of unique kmers, unless the taxon in question had > 90% completeness, following Danko et al. [32]. The taxonomic feature tables from KrakenUniq were imported into the phyloseq R package [36] for further analyses.

Several taxa were identified in negative controls (two reagent negatives, two SASS filter negatives, and one Bobcat filter negative). We assessed these potential contaminants by comparing their genomic coverage—which is available by the unique Kmer statistic in KrakenUniq—in negative and actual samples. For any given taxa, we required that actual air samples had 20% higher genomic coverage than in the negative samples. The rationale for this approach hinges on the assumption that if a taxonomic hit in actual samples is a pure contaminant, the taxonomic coverage should not exceed that in negative controls—in fact, with knockdown effects one would expect coverage to be lower in actual samples, all other factors being equal. The 20% higher coverage is thus conservative. Taxa identified in negative controls that did not meet the 20% higher coverage criterion were removed prior to further analyses.

Rarefaction curves were evaluated for the outdoor and subway datasets separately before rarefying both to their respective minimum read depths (i.e., species-level assigned reads). Relative abundances for the top 20 species were plotted in sample pairs (collected with the Bobcat and SASS samplers). Observed richness and Shannon’s diversity index calculated at the species level, were compared for the two air samplers using a two-sample Kolmogorov-Smirnov test, which compares empirical cumulative distributions. Beta diversity was evaluated at the species level by the analysis of distance matrices (Bray–Curtis dissimilarity and Jaccard index) with PERMANOVA (“adonis” function in the vegan R package [38]). Distance matrices were ordinated with PCoA for visualization. Heatmaps were used for comparisons of all observed species within sample pairs. Lastly, species that were only identified in samples collected by either SASS or Bobcat were presented in separate heatmaps.

#### Accession numbers

The sequence data has been deposited in the NCBI Sequence Read Archive under Bioproject ID# PRJNA527324 (https://www.ncbi.nlm.nih.gov/sra/PRJNA527324) and PRJNA561080 (https://www.ncbi.nlm.nih.gov/sra/PRJNA561080).

## Results

### Aerosol chamber-based sampling efficiency evaluation

#### Sass 3100

**Fig. 2:**
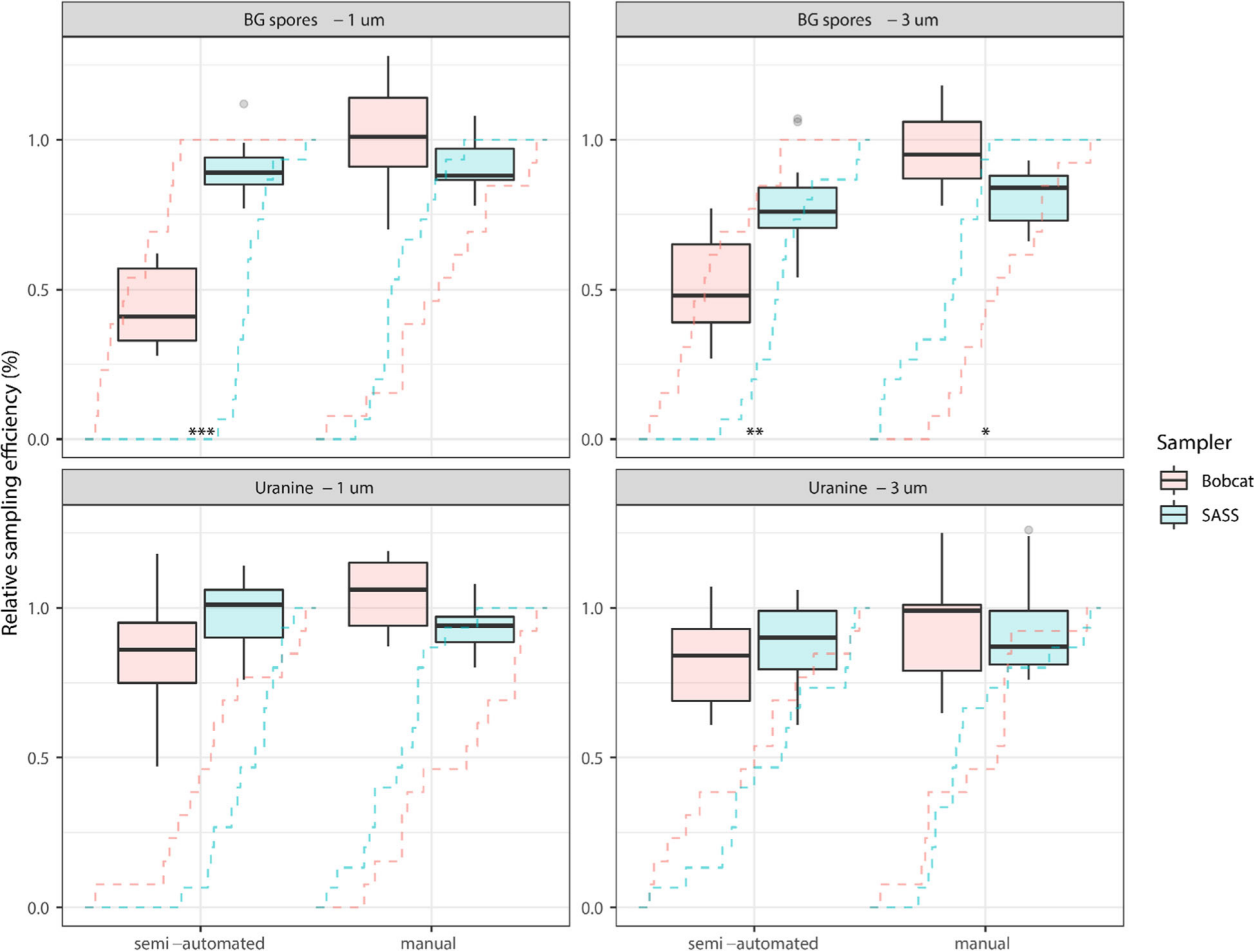
Physical sampling efficiencies. Sampling efficiencies (%) for SASS and Bobcat relative to gelatin reference filters. Sampling efficiency was determined with a common manual filter extraction procedure and sampler-specific semi-automated filter extraction procedures. Aerosol chamber testing was performed with 1 μm (left panels) and 3 μm (right panels) aerosol particles containing Uranine (bottom panels) as a fluorescent tracer and BG spores (top panels) as a biological tracer. The number of samples analyzed for each condition was *N* = 15 for SASS and *N* = 13 for Bobcat. Empirical cumulative distribution functions of sampling efficiencies are overlain the boxplots, and significance codes from Kolmogorov-Smirnov tests are indicated at the bottom of each sampler comparison (codes: *** *P* < 0.001, ** *P* < 0.01, * *P* < 0.05)

With manual filter extraction, the sampling efficiency based on Uranine was 93 ± 7% (1 μm) and 93 ± 16% (3 μm), while the efficiency based on BG spores was 91 ± 8% (1 μm) and 81 ± 9% (3 μm; Fig. 2). For semi-automated filter extraction, the sampling efficiency based on Uranine was 98 ± 11% (1 μm) and 88 ± 13% (3 μm), while the efficiency based on BG spores was 90 ± 8% (1 μm) and 78 ± 14% (3 μm; Fig. 2). No significant differences (*P* > 0.38) in sample recovery were found between manual and semi-automated filter extraction.

#### ACD-200 bobcat

With manual filter extraction, the sampling efficiency based on Uranine was 104 ± 11% (1 μm) and 92 ± 16% (3 μm), while the efficiency based on BG spores was 101 ± 16% (1 μm) and 97 ± 11% (3 μm; Fig. 2). The sampling efficiency for semi-automated filter extraction based on Uranine was 87 ± 19% (1 μm) and 82 ± 15% (3 μm), while the efficiency based on BG spores was 44 ± 12% (1 μm) and 51 ± 16% (3 μm; Fig. 2). Significant differences in sample recovery were found between manual and semi-automated filter extraction for 1 μm BG spores (101 ± 16% vs 44 ± 12%; *P* > 0.001) and 3 μm BG spores (97 ± 11% vs 51 ± 16%; *P* > 0.001; Fig. 2).

#### Air sampler comparison

The end-to-end sampling efficiency was > 80% under all four test conditions for both samplers with manual filter extraction. The sampling efficiency of SASS and Bobcat with manual extraction was significantly different for 3 μm BG spores (91 ± 8% vs 101 ± 16%; Fig. 2; *P* = 0.03). No significant differences were observed for 1 μm Uranine (93 ± 7% vs 104 ± 11%; *P* = 0.09), 3 μm Uranine (93 ± 16% vs 92 ± 16%; *P* = 0.63) and 1 μm BG spores (81 ± 9% vs 97 ± 11%; *P* = 0.12).

### Evaluation of sampling performance in outdoor air and on subway stations

#### Total biomass

**Fig. 3:**
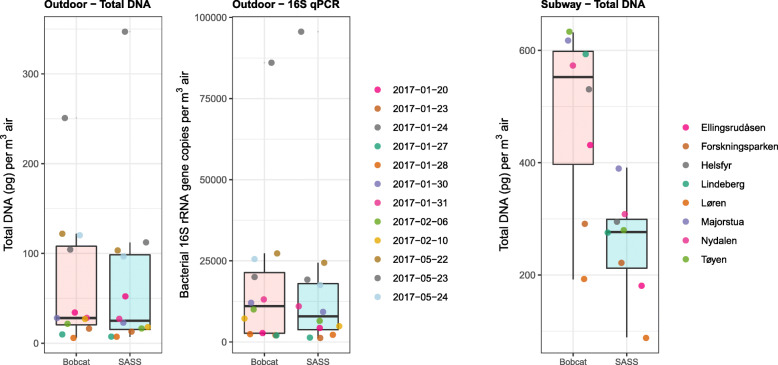
Total DNA and bacterial 16S rRNA gene copy yields. Total DNA and bacterial 16S rRNA gene copy yields in outdoor air samples (left panels), and total DNA yield from subway air samples (right panel). Total DNA yield was expressed as picograms per cubic meter of air. Bacterial 16S rRNA gene copy yield was expressed as bacterial 16S rRNA gene copies per cubic meter of air. Jittered data points, overlain the boxplots, are color coded by sample pair

DNA yield (pg) per cubic meter of sampled air was not significantly different (*P* = 0.99) between SASS (69 ± 92 pg m^− 3^) and Bobcat (64 ± 70 pg m^− 3^) in the semi-suburban outdoor environment (Table S2 and Fig. 3). However, for the complex subway environment there was a significant difference (*P* = 0.02) between SASS (255 ± 91 pg m^− 3^) and Bobcat (483 ± 153 pg m^− 3^; Table S3 and Fig. 3).

#### Bacterial biomass

Bacterial 16S rRNA gene copy yield per cubic meter of sampled air was not significantly different (*P* = 0.99) between SASS (16,456 ± 24,935 copies m^− 3^) and Bobcat (17,552 ± 22,354 copies m^− 3^), suggesting comparable sampling efficiency for total bacterial biomass when sampling an outdoor environment (Table S2 and Fig. 3).

#### SMS

After quality trimming and removal of human and PhiX reads, there remained 208 M reads in the outdoor dataset (average: 8.6 M; range: 5.9–16.3 M; Table S2) and 40 M in the subway dataset (average: 2.5; range: 1.25–2.99; Table S3). Both datasets were rarefied to the lowest number of species assigned reads, 138,321 and 148,141 respectively (Figure S1). Two and 12 species were flagged as potential contaminants, and were removed from the outdoor and subway datasets respectively. After contaminant removal, the outdoor dataset contained 460 species, while the subway dataset consisted of 606 species.

**Fig. 4:**
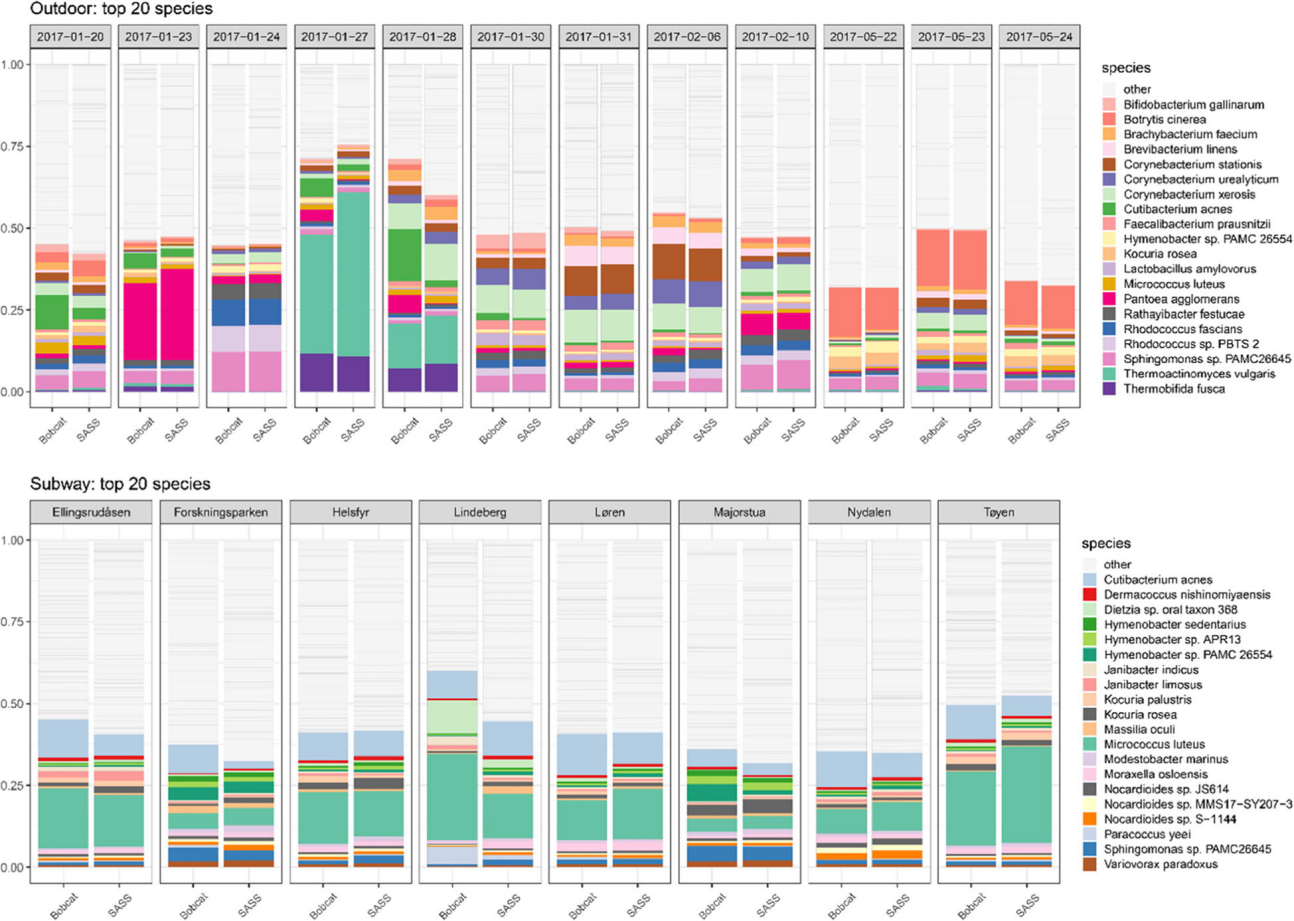
Taxonomic overview (top 20 most abundant species). The top 20 most abundant species shown for sample pairs from outdoor and subway sampling campaigns. Pairs were sampled side-by-side with SASS and Bobcat air samplers

**Fig. 5:**
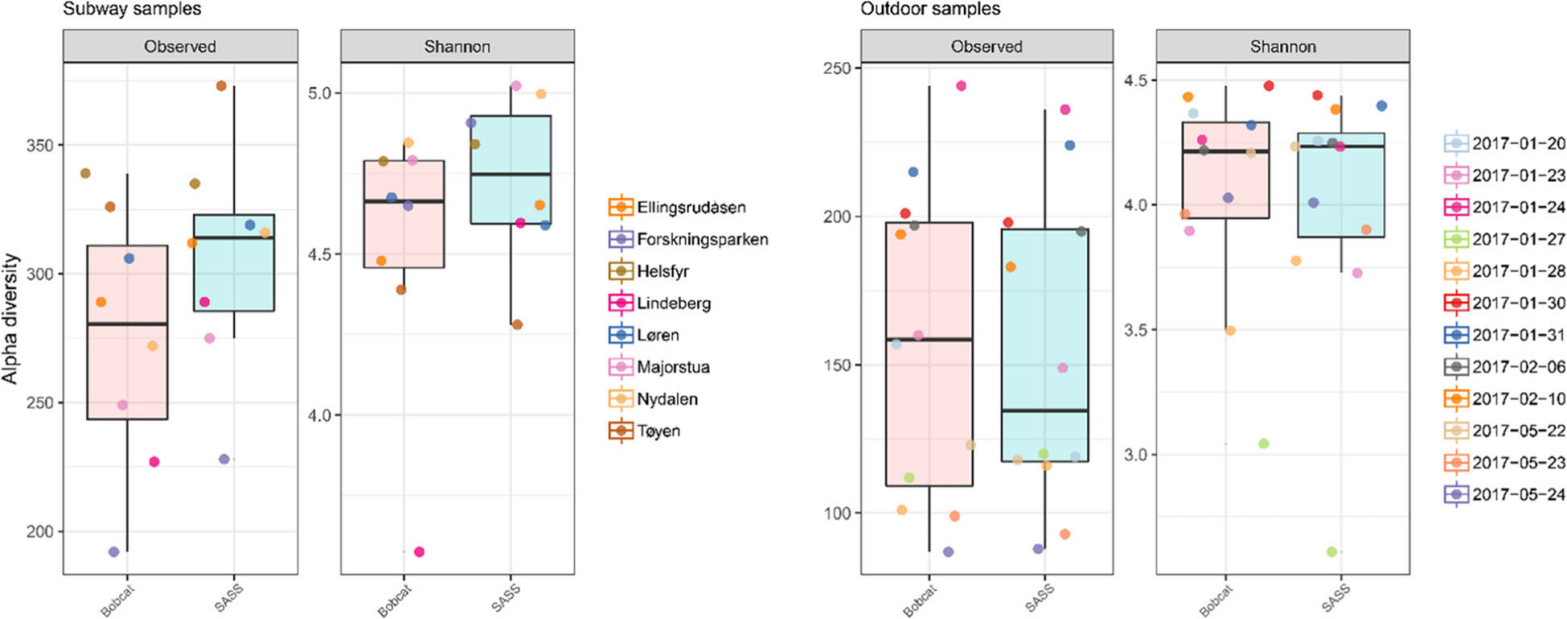
Alpha diversity distribution. Alpha diversity distribution (Observed number of species and Shannon’s diversity index) for subway station (left panels) and outdoor (right panels) samples collected with SASS and Bobcat air samplers. Sample pairs, i.e., samples collected side-by-side with both sampler types, are color coded

**Fig. 6:**
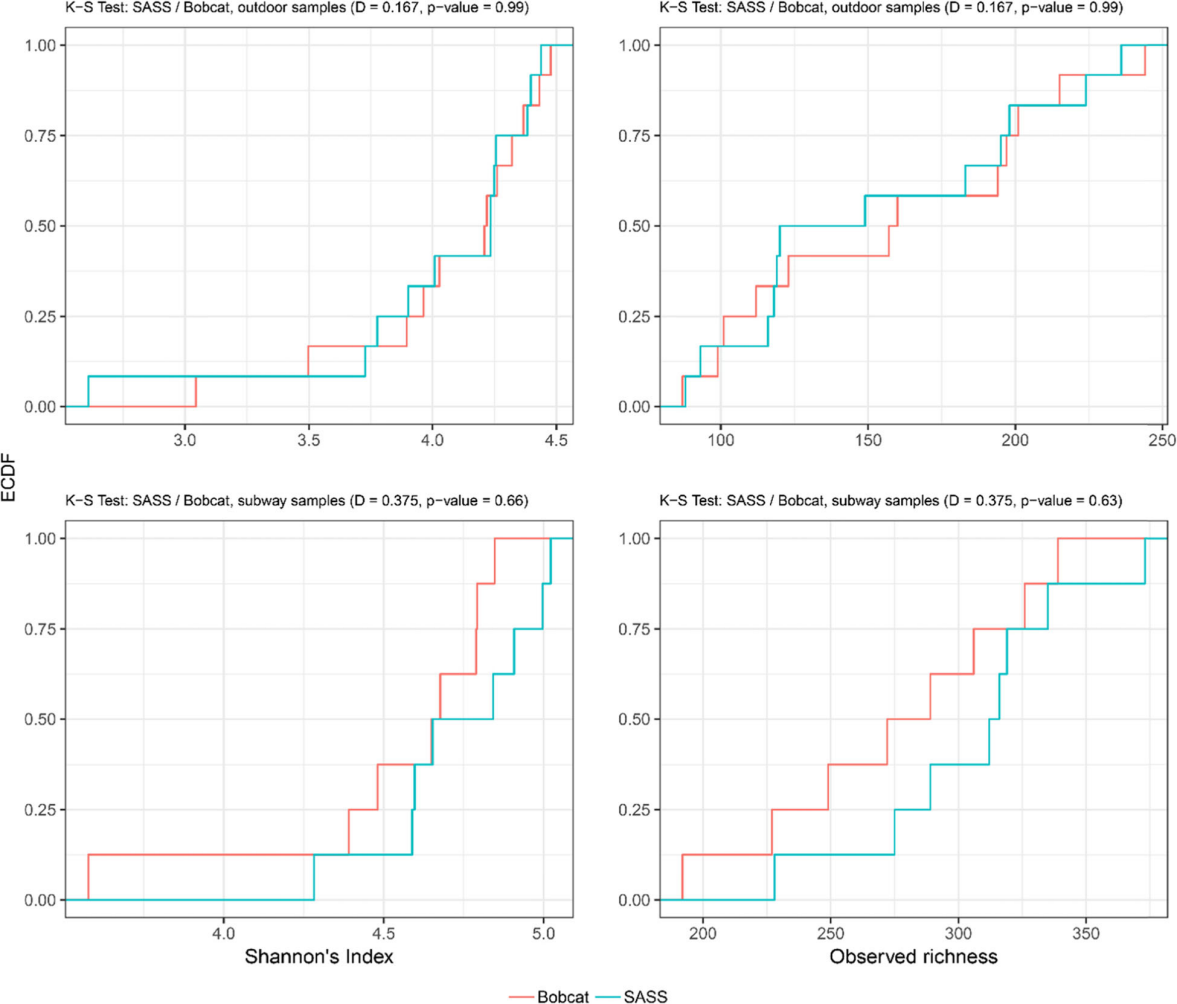
Empirical cumulative distribution functions of alpha diversity and associated Kolmogorov-Smirnov tests. Alpha diversity cumulative functions for outdoor (top panels) and subway (bottom panels) air samples collected with SASS and Bobcat. The Kolmogorov-Smirnov tests evaluate the null-hypothesis that samples are drawn from the same distribution, where significant results (*P* > 0.05) indicate different distributions

**Fig. 7:**
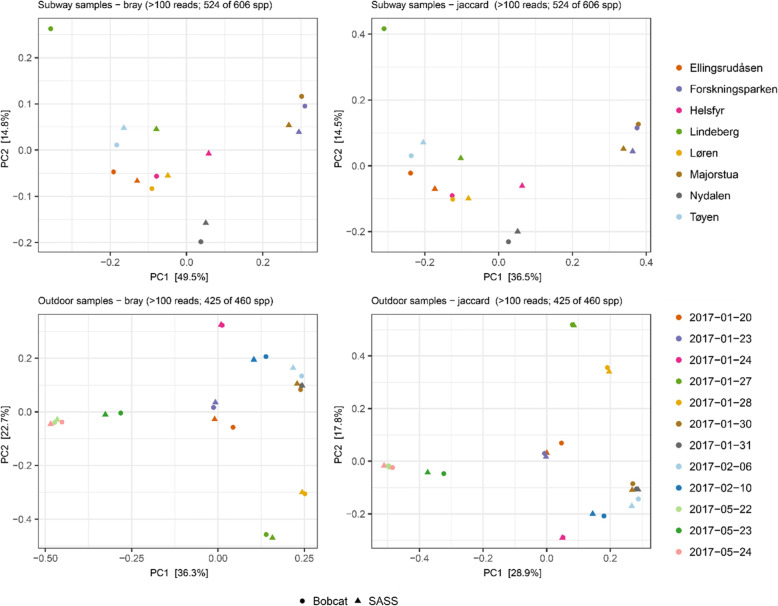
Beta diversity cluster plots. Cluster plots (PCoA) of Bray–Curtis dissimilarity and Jaccard Index for subway (top panels) and outdoor (bottom panels) air sample pairs collected with SASS and Bobcat

**Fig. 8:**
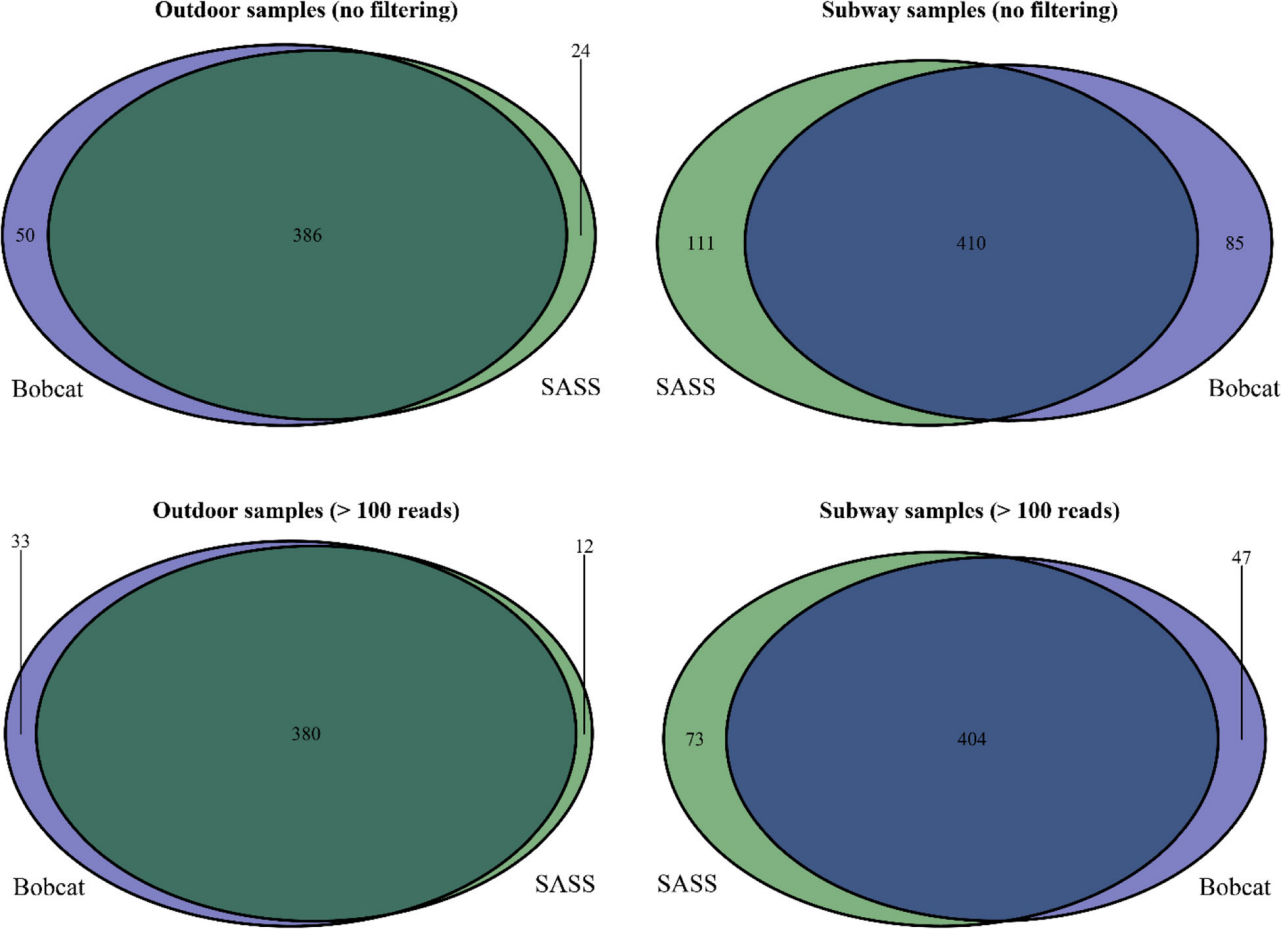
Venn diagram of species distributions. Species presence in air samples collected with SASS and Bobcat. Top panels: all species included. Bottom panels: only species with > 100 reads included

**Fig. 9:**
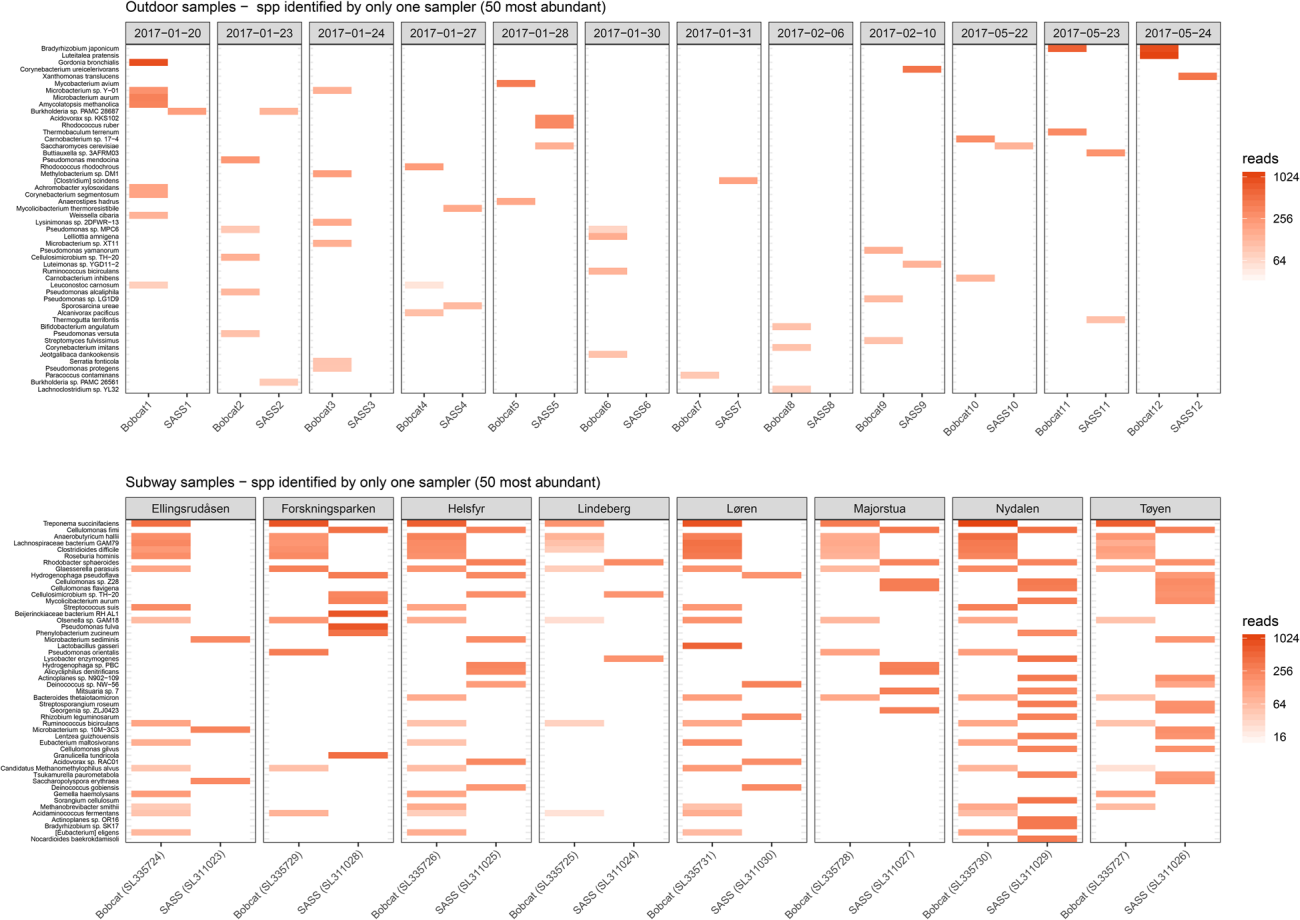
Heatmap of species only identified by one air sampler. Species (top 50 most abundant) only identified in outdoor (top panel) and subway (bottom panel) air samples from either SASS or Bobcat

The top 20 most abundant species showed similar distributions within sample-pairs in both the outdoor and subway dataset (Fig. 4). Alpha diversity (observed diversity and Shannon’s diversity index; Fig. 5 and Table S2) was not significantly different (*P* > 0.63; Fig. 6) between SASS and Bobcat samples for either dataset, i.e., comparable microbial diversity estimates were obtained when sampling complex environmental bioaerosols. PERMANOVA tests revealed that the observed beta diversity (Bray–Curtis dissimilarity) was significantly explained by sample pairs (outdoor dataset: *R*^*2*^ = 0.97, *P* = 0.001; subway dataset: *R*^*2*^ = 0.78, *P* = 0.001), while air sampler type was not significant (outdoor dataset: *R*^*2*^ < 0.01, *P* = 0.99; subway dataset: *R*^*2*^ = 0.10, *P* = 0.15). The same tests using Jaccard Index gave nearly identical values. PCoA cluster plots (Fig. 7) showed that outdoor sample pairs (SASS and Bobcat samples) clustered very tightly, whereas subway sample pairs were more diverged. The higher variability within subway samples were further confirmed by Venn diagram plotting, which revealed that a higher proportion of species were unique to either sampler type in the subway dataset (Fig. 8). Lastly, we isolated species identified in samples from only one of the two samplers. In the outdoor dataset, these species were largely restricted to single samples, whereas for subway samples a number of species only identified by one sampler were present in several samples (Fig. 9). In addition to a heatmap of all species for both outdoor and subway (Figure S2), we also produced a heatmap of species that were only identified by one sampler type in both outdoor and subway data (Figure S3). Of these 11 species, 10 were unique to the same sampler type in both outdoor and subway samples.

## Discussion

The evaluated high-volume electret filter air samplers (SASS 3100 and ACD-200 Bobcat) consistently achieved end-to-end sampling efficiencies > 80% when combined with manual filter extraction under all test conditions in the aerosol chamber-based evaluation. These results suggest both air samplers are capable of effective aerosol biomass sampling and also capable of retaining a reliable quantitative association between the recovered sample and the sampled air environment. In the real-world environment, both samplers recovered sufficient aerosol biomass amounts for direct SMS and gave rise to comparable diversity estimates and taxonomic distributions for the most abundant species. Some distinct differences were nevertheless observed at the species level. Previous air sampler comparisons have shown conflicting diversity patterns [3, 23, 39], which could partially be attributed to differences in aerosol collection and sample recovery principles. Without a common consistency and understanding of how differences between methodologies arise, it is difficult to establish an aerosol microbiome baseline for real-world environments. By performing pairwise comparisons with different air samplers in real-world environments we could better 1) predict bias introduced by different samplers, and 2) understand how results can be compared across studies utilizing different air samplers. In the long run this could ultimately help us define a better aerosol microbiome baseline.

The end-to-end sampling efficiency of an air sampler does not only depend on the collection efficiency, but also on how efficiently collected particles can be recovered from the sampler and sample [40]. Therefore, the recommended sampler-specific, semi-automated filter extraction procedure for the SASS and Bobcat was compared to a common manual procedure to assess the impact on recovery efficiency. The results showed that SASS had similar recovery efficiencies for semi-automated and manual extraction, while Bobcat had significantly reduced recovery efficiencies for semi-automated compared to manual extraction (Fig. 2). Interestingly, the recovery efficiency reduction observed for Bobcat was only significant for BG spores (Fig. 2). This finding may reflect differences in how efficiently the involved surfactants de-agglomerates or prevents agglomeration of bacterial spores, or it may suggest that bacterial spores are more difficult to recover from electret filters during liquid extraction, which could reflect the hydrophobic surface properties of bacterial spores [41]. It may therefore be speculated that the surfactant used in Bobcat Rapid Filter Elution kits (0.075% Tween-20) was unable to completely recover BG spores from the filters, while the surfactant used in SASS Extraction kits (0.05% Triton X-100) achieved a more efficient recovery. This observation highlights the importance of carefully selecting an extraction liquid formulation, including surfactant type and concentration, which allows for efficient biomass recovery from electret filters regardless of type and state of the captured microorganisms. Thus, only the common manual filter extraction procedure was used when comparing the two air samplers in this study.

SASS and Bobcat were subjected to aerosol chamber-based evaluation to benchmark and compare their physical sampling efficiency as a generic proxy for total aerosol biomass sampling efficiency. The results revealed that Bobcat had a significantly higher sampling efficiency for 3 μm BG spores (*P* = 0.03), and was borderline significant for 1 μm particles (BG spores, *P* = 0.12; Uranine, *P* = 0.09) using the manual filter extraction procedure. Although the two air samplers and also the electret filters they rely on are similar, they are not identical. The observed sampling efficiency differences may therefore potentially be explained by differences in the design and construction of the air samplers (e.g. inlets) and/or the electret filters, but further investigations would be required to conclude on this matter. However, both samplers demonstrated that they were capable of efficient and quantitative volumetric sampling of total aerosol biomass, with sampling efficiencies > 80% under all test conditions using manual filter extraction. The test materials and particle sizes used in the chamber-based evaluation only reflect a small portion of the true complexity of environmental aerosol biomass, and this should be taken into consideration when interpreting the observed difference between the two samplers. In future studies, it would be interesting to expand the range of test materials and leverage real-world aerosol microbiome knowledge to cover a broader range of environmental bioaerosols, both in terms of additional types of microorganisms and particle sizes. This could help us identify and better understand potential differences in end-to-end sampling efficiency (collection and/or recovery efficiency) due to material or particle size. However, at current time, the carefully controlled environment do not fully reflect real-world sampling conditions and it is therefore important to include application-oriented performance evaluations in relevant environments [4].

To this end, the second stage of the performance evaluation in this work involved side-by-side sampling with SASS and Bobcat in an outdoor semi-suburban environment (Kjeller, Norway) and on subway stations (Oslo, Norway). Increasing the sampling time is a common strategy to ensure capture of sufficient biomass for downstream applications, but it will also negatively impact (reduce) the temporal resolution. Based on past sampling experience in Norway, the subway environment in Oslo typically allows for a shorter sampling time than the outdoor semi-suburban ambient environment in Kjeller, especially in the winter season. A longer sampling time was therefore used in this study when sampling in the outdoor ambient environment (6–8 h) than in the subway environment (30 min). However, the gelatin filters used for benchmarking purposes in the chamber-based evaluation was not deemed feasible to use as a reference in conjunction with low biomass environments and SMS due to the low flowrate. The performance evaluation involved direct comparison of total DNA and bacterial 16S rRNA gene copy yields per cubic meter of sampled air (Fig. 3), and SMS-based aerosol microbiome profiles (Figs. 4, 5, 6, 7, 8 and 9) between paired air samples. The results revealed that SASS and Bobcat delivered highly similar sampling performance when sampling semi-suburban outdoor ambient air, both in terms of amount (Fig. 3) and type (Figs. 4, 5, 6 and 7) of biomass collected. The results for samples collected in eight different subway stations revealed a significant difference in total DNA yield per cubic meter of sampled air, with Bobcat exhibiting higher yields (Fig. 3), and more within-pair taxonomic variability (Fig. 7). However, when taking into account that SASS (300 LPM) was operated at a higher flowrate than Bobcat (200 LPM), there was no significant differences in absolute DNA yield (*data not shown*), which in the context of direct SMS probably would be the most important yield parameter. While taxonomic distributions (20 top most abundant species; Fig. 4) and comparisons of alpha diversity (Figs. 5 and 6) showed similar patterns between sampler types, there was weaker clustering of sampler pairs from subways (Beta diversity; Fig. 7). This may be due to larger variability among sample pairs collected outdoors, i.e., if all sample pairs from different subway stations are by comparison similar, one would expect less clustering even if there are small within-pair differences. Also, larger micro-scale variability in subway air as compared to outdoor or the different sampling durations may partly explain these findings. There were no significant differences in Bray Curtis dissimilarity or Jaccard index among samples that were collected with either SASS and Bobcat; however, while sampler type explained only 0.5% of the variation in outdoor samples (*P* = 0.99), it explained 10.4% in subway samples (*P* = 0.14). While higher micro-scale variability in subways, or generally larger within-system variability outdoors, may explain much of these results, we also observed consistent identification of certain species by only one sampler (Fig. 9). These patterns were most pronounced in subway samples, but some species showed the same sampler-specific representation across the two independent datasets (outdoors and subway stations; Figure S3). These results indicate that a few species are only detectable through either sampler which is noteworthy given the distinct similarities in terms of collection technology/principle between these two samplers. To identify if the weaker clustering seen in the subway environment is a result of micro-scale variability, future studies should investigating stochastic effects when using identical samplers. This could provide us with more in depth knowledge on how to interpret results obtained from multiple air samplers.

The outcome of this work contributes to improved understanding of air sampler selection for SMS-based aerosol microbiome research, especially in low biomass environments. Selection of air sampling equipment for use in aerosol microbiome research ultimately depends on many factors, including study objective, research questions, size and type of targeted bioaerosol particles, and practical considerations. Nevertheless, to ensure that the results from aerosol microbiome investigations can be reliably compared with the results from other studies, which is fundamental to ensure scientific progress, the use of air samplers with well-defined and benchmarked performance characteristics should be repeatedly emphasized as a communal responsibility.

## Conclusions

The aerosol chamber-based performance evaluation showed that SASS and Bobcat consistently achieved end-to-end sampling efficiencies > 80% when adopting a manual filter extraction procedure. This suggest that both samplers are capable of effective aerosol biomass sampling and also of retaining a reliable quantitative association between the recovered sample and the sampled air environment. The performance evaluation in real-world environments demonstrated that both samplers were capable of collecting sufficient amounts of aerosol biomass for SMS, even with a 30-min sampling time. The SMS-based aerosol microbiome comparison showed that the diversity estimates and taxonomic distributions for the most abundant species were highly comparable between the two samplers. Nevertheless, some distinct differences in microbiome profiles where identified, particularly for subway samples. This suggest that these samplers, which are based on the same collection technology/principle, should not be treated as directly interchangeable. Taken together, our findings nevertheless suggest that both samplers are well suited for use in aerosol microbiome research and that meaningful comparisons of results should be possible. We hope that our study may inspire the aerosol microbiome community to continue emphasizing the need for increased performance benchmarking, harmonization and standardization of air sampling procedures, including air sampling equipment, sampling protocols and downstream sample processing and analytics. The lack of such is still an important and unresolved challenge that makes it hard to reliably compare and extrapolate results between aerosol microbiome studies that have used different methodology. This challenge ultimately limits scientific progress and thus deserves increased attention [4, 21, 42–48].

## Supplementary information

**Additional file 1: Supplementary Text 1.** Aerosol chamber-based benchmarking of reference samplers. **Supplementary Text 2.** Aerosol chamber-based evaluation of SASS 3100 sampling efficiency as a function of sampling flowrate. **Figure S1.** Rarefaction curves for the outdoor and subway datasets. **Figure S2.** Heatmaps showing transformed read counts for all species identified in the outdoor and subway datasets. **Figure S3.** Heatmaps showing the 12 species that were only detected in samples from one sampler in both the outdoor and subway dataset. **Table S1.** Particle size distribution for 1 and 3 μm aerosol particles used for the aerosol chamber-based sampling efficiency evaluation. **Table S2.** Metadata outdoor air sampling campaign. **Table S3.** Metadata subway air sampling campaign.

## Data Availability

The sequence data has been deposited in the NCBI Sequence Read Archive under Bioproject ID# PRJNA527324 (https://www.ncbi.nlm.nih.gov/sra/PRJNA527324) and PRJNA561080 (https://www.ncbi.nlm.nih.gov/sra/PRJNA561080).
