# Supplementary material for: Performance evaluation of high-volume electret filter air samplers in aerosol microbiome research
Source: Environ Microbiome. 2020 Jul 28;15:14. doi: 10.1186/s40793-020-00362-x (PMC8067322; doi:10.1186/s40793-020-00362-x)
Supplement: Supplementary file 1 — Additional file 1: Supplementary Text 1. Aerosol chamber-based benchmarking of reference samplers. Supplementary Text 2. Aerosol chamber-based evaluation of SASS 3100 sampling efficiency as a function of sampling flowrate. Figure S1. Rarefaction curves for the outdoor and subway datasets. Figure S2. Heatmaps showing transformed read counts for all species identified in the outdoor and subway datasets. Figure S3. Heatmaps showing the 12 species that were only detected in samples from one sampler in both the outdoor and subway dataset. Table S1. Particle size distribution for 1 and 3 μm aerosol particles used for the aerosol chamber-based sampling efficiency evaluation. Table S2. Metadata outdoor air sampling campaign. Table S3. Metadata subway air sampling campaign. [file 40793_2020_362_MOESM1_ESM.pdf]

Supplementary Text 1. Aerosol chamber-based benchmarking of reference samplers.

Aim

Benchmark three reference air samplers (SKC BioSampler, gelatin filters, and isopore membrane filters) commonly used in aerosol chamber testing.

Method

The sampling efficiency of isopore membrane filters (Millipore DTTP0047, pore size 0.6 µm), gelatin filters (Sartorius 12602-47-ALK) and the SKC BioSampler (SKC 225-9595) was tested in the aerosol chamber using FluoSpheres (1 µm; 3.7 µm; ThermoFisher F8823 and F8859) and polystyrene latex beads (8 µm; Merck 78511). The spray solution was made by mixing 1 µm (50 µl), 3.7 µm (1 ml) and 8 µm (2 ml) beads in MQ water to a final volume of 10 ml. A Hudson medical nebulizer, generated aerosols for 3 minutes before aerosols were allowed to homogenize for 1 minute. Aerosol collection with gelatin and isopore membrane filters was performed as described in "Aerosol collection" with a flowrate of 15 LPM. The SKC BioSampler was filled with 20 ml PBSTA buffer and sampled at 12.5 LPM. The sampling time was set to 10 minutes, and the experiments were repeated six times for each particle size. After sampling, filters were treated as described in "Manual filter extraction", except the isopore membrane filters were vortexed at maximum speed for 30 seconds to wash microspheres off the filter. Sampling buffer from the SKC BioSampler was transferred to a 50 ml PP tube. Quantification was performed with a BD Accuri C6 flow cytometer (BD Biosciences, NJ, USA).

Results

Gelatin filters were used as a reference, and relative sampling efficiency (%) for SKC BioSampler and isopore membrane filters was therefore expressed relative to gelatin filters. The sampling efficiency for isopore membrane filters was 98-100% for all particle sizes, and 72-86% for the SKC BioSampler (Supplementary Text 1 Table 1).

The sampling efficiency was significantly different between isopore membrane filters and SKC BioSampler for all particle sizes (1 µm, P = 0.009; 3.7 µm, P = <0.001; 8 µm, P = 0.035).

Supplementary Text 1 Table 1. Sampling efficiencies (%) for isopore membrane filters and SKC BioSampler relative to gelatin filters.

	**1 µm**	**3.7 µm**	**8 µm**
**Isopore membrane filters**	98±8%	98±8%	100±13%
**SKC BioSampler**	86±2%	72±7%	82±9%

Supplementary Text 2. Aerosol chamber-based evaluation of SASS 3100 sampling efficiency as a function of sampling flowrate.

Aim

Evaluate sampling efficiency of SASS 3100 at different sampling flowrates to better gauge the flowrate-dependency of the observed sampling efficiency.

Method

The sampling efficiency of the SASS 3100 was evaluated at four different sampling flowrates (50, 100, 200 and 300 LPM). The aerosol chamber-based sampling efficiency evaluation included two particle sizes (1 and 3 µm) of BG spores and Uranine as described in "Aerosol chamber-based sampling efficiency evaluation" using manual filter extraction. Four SASS 3100 samplers were used to sample simultaneously at each flowrate 50, 100, 200 and 300 LPM using gelatin filters as a reference. The samplers rotated on sampling at the different flowrates, and a minimum of five experiments were performed at each flowrate under all four test condition. Quantitation was performed as described in "Plate count analysis" and "Fluorimeter analyses".

Results

The sampling efficiency (%) relative to gelatin reference filters was >87% under all test conditions (Supplementary Text 2 Table 1). Based on ANOVA tests, the observed sampling efficiency was not significantly different between any of the evaluated sampling flowrates.

Supplementary Text 2 Table 1. Relative sampling efficiency (%) at different flowrates with SASS 3100.

		50 LPM	100 LPM	200 LPM	300 LPM
**1 µm**	**BG**	93±6	96±14	95±14	87±11
	**Uranine**	90±5	96±8	93±16	87±2
**3 µm**	**BG**	92±11	88±8	94±12	88±12
	**Uranine**	91±9	103±7	100±11	88±6

Figure S1. Rarefaction curves for the outdoor (A) and subway (B) datasets. Dashed vertical lines indicate the read depth (reads assigned to the species level) to which all samples were rarefied.

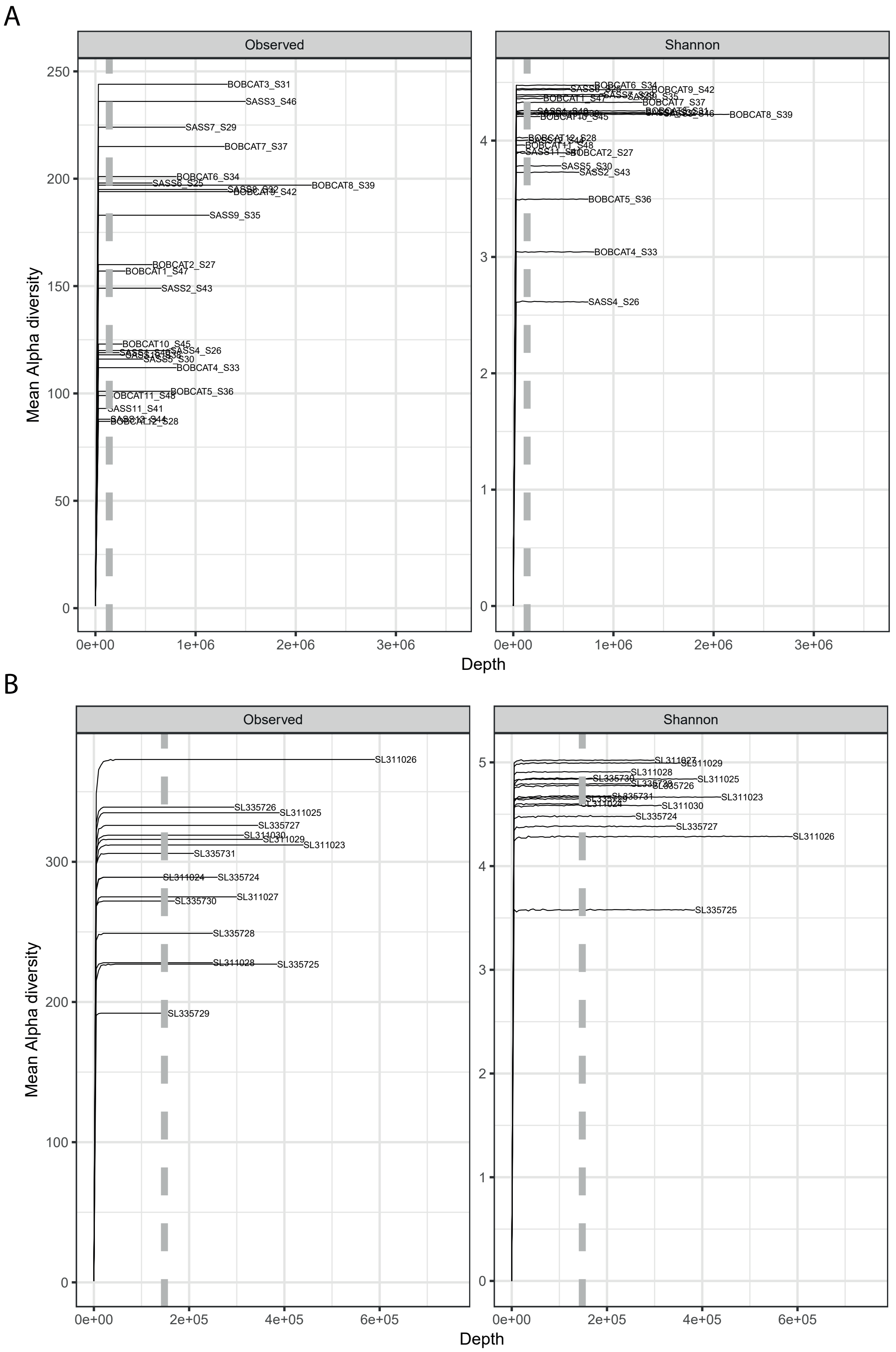

Figure S2. Heatmaps showing transformed read counts for all species identified in the outdoor (460 spp) and subway (606 spp) datasets.

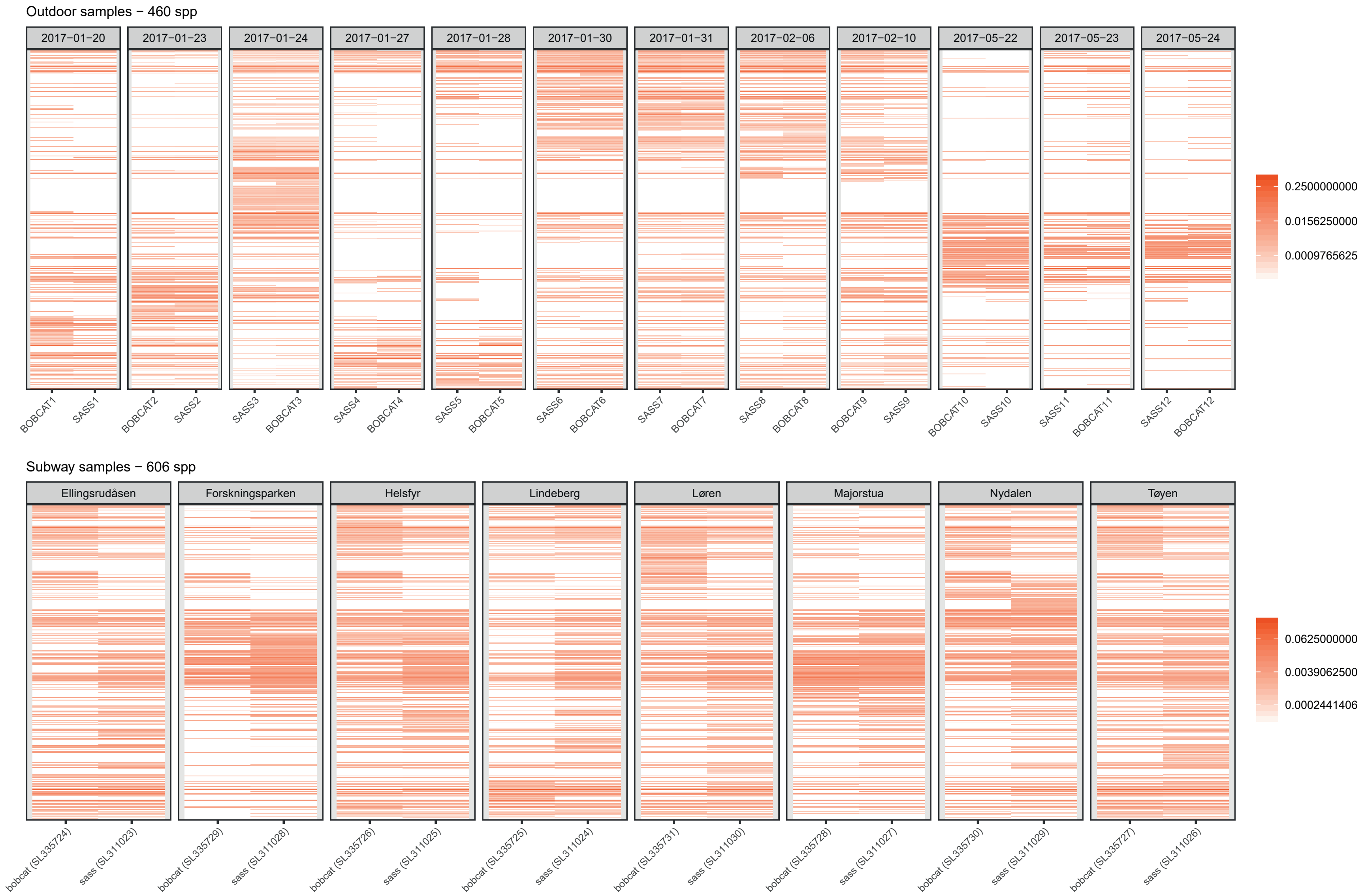

Figure S3. Heatmaps showing the 12 species that were only detected by a single sampler in both subway and surface, where some were detected by the same sampler in both data sets and some with different samplers.

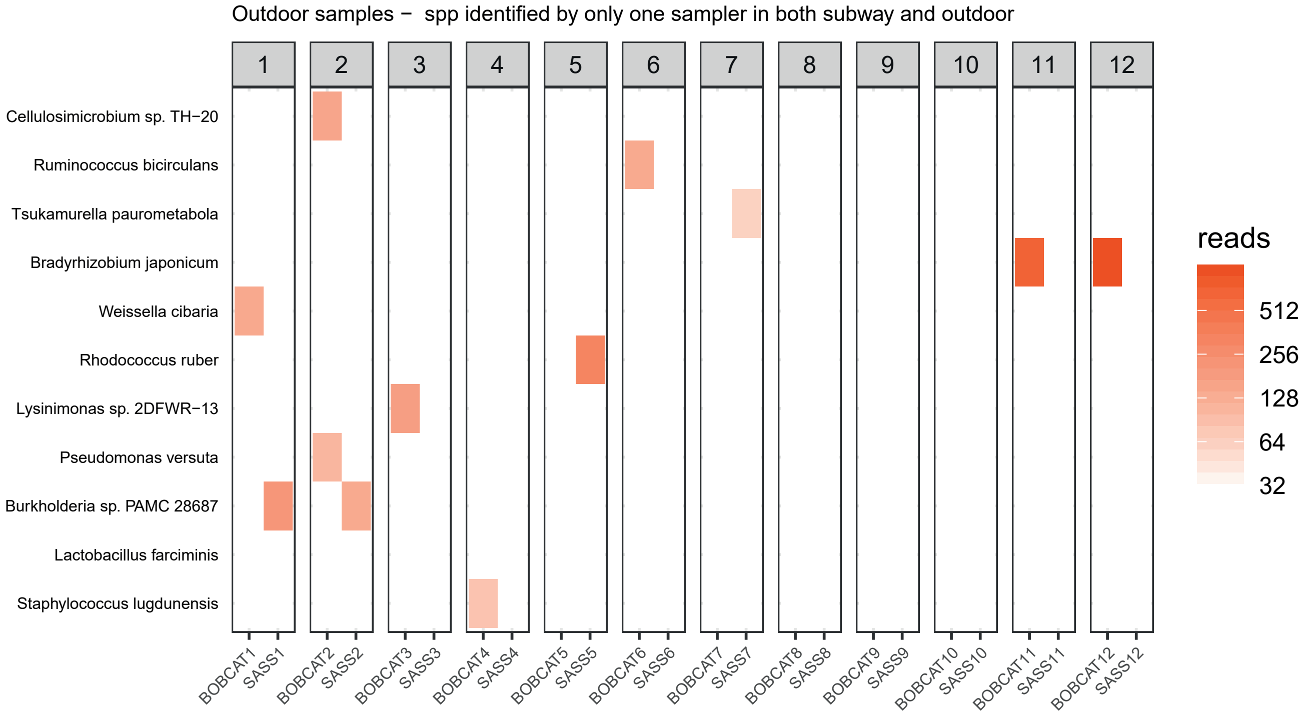

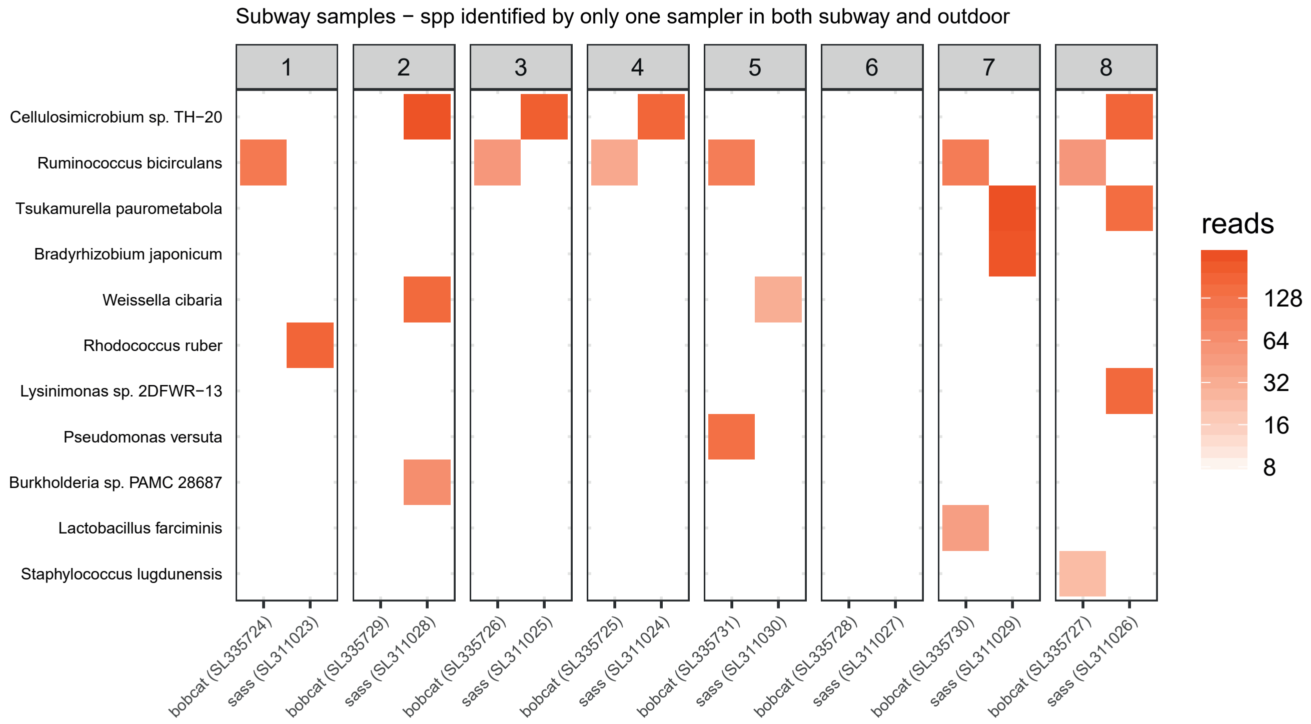

Table S1. Particle size distribution for 1 and 3 µm particles (droplet nuclei) aerosolized using 120- and 48-kHz ultrasonic atomizer nozzles, respectively, from spray liquid containing mixture of *Bacillus atrophaeus* (BG) spores and Uranine. Particle size distribution was calculated based on APS 3321 aerodynamic particle sizer measurements from five or more aerosol experiments and reported as mean (± standard deviation) count median aerodynamic diameter (µm) and geometric standard deviation (unitless).

**Aerosol generator (targeted particle size)**	**Particle size distribution**	
	**Count median aerodynamic diameter (CMAD) (mean ± standard deviation, µm)**	**Geometric standard deviation (GSD) (mean ± standard deviation, unitless)**
120-kHz ultrasonic atomizer nozzle (~1 µm particles)	1.07 ± 0.01	1.41 ± 0.01
48-kHz ultrasonic atomizer nozzle (~3 µm particles)	2.99 ± 0.05	1.26 ± 0.01

Table S2. Metadata outdoor air sampling campaign. Twelve paired air samples were collected side-by-side with SASS 3100 and ACD-200 Bobcat in a semi-suburban outdoor environment (Kjeller, Norway, 59.976540N, 11.048691E) between January-May 2017. Air sampling was performed for a period of 6-8 hours with the samplers mounted on tripods with an inlet height of ~1.5 meters. SASS and Bobcat were operated at a sampling flowrate of 300 and 200 liters of air per minute (LPM), respectively.

**Date**	**Time of day**	**Meteorological conditions**					**SASS 3100**							**ACD-200 Bobcat**						
							**Yield**		**Shotgun metagenomic sequencing (read statistics)**			**Alpha Diversity (species)**		**Yield**		**Shotgun metagenomic sequencing (read statistics)**			**Alpha Diversity**	
		**Temp (°C)**	**RH (%)**	**Precipitation (mm)**	**Wind speed (m/s)**	**Wind direction (wind from)**	**Total DNA (pg m^{-3})**	**Bacterial 16S rRNA gene (copies m^{-3})**	**Raw reads (150 bp, paired)**	**Quality filtered reads (≥Q20, ≥100 bp, PhiX and human removed, paired)**	**Classified reads (KrakenUniq, NCBI RefSeq database, paired)**	**Shannon Index**	**Observed Richness**	**Total DNA (pg m^{-3})**	**Bacterial 16S rRNA gene (copies m^{-3})**	**Raw reads (150 bp, split pairs)**	**Quality filtered reads (≥Q20 and ≥100 bp), PhiX and human removed**	**Classified reads (KrakenUniq, NCBI RefSeq database)**	**Shannon Index**	**Observed Richness**
20.01.2017	08:20-15:20	6.3±1,9	60±6	0	1.9±0.7	West	52	4 319	12 337 993	7 823 604	685 494	4,256	119	34	2 750	10 729 895	6 837 695	717 589	4,367	157
23.01.2017	08:20-16:20	-0.8±1.3	73±8	0	1.2±1.3	South	13	1 255	12 424 466	7 876 624	1 220 753	3,727	149	16	2 085	10 581 444	6 750 599	1 094 393	3,896	160
24.01.2017	09:40-15:40	-6.0±2.3	93±2	0	0.0±0.0	South	347	95 601	13 500 418	8 679 882	2 395 128	4,234	236	251	86 041	11 074 628	7 364 197	2 087 043	4,261	244
27.01.2017	08:10-16:10	0.4±0.2	97±2	0	0.5±0.6	South-West	7	1 340	9 567 532	6 427 590	1 367 842	2,610	120	10	2 039	12 797 639	8 680 209	1 648 233	3,044	112
28.01.2017	11:20-17:50	0.2±0.3	87±3	0	1.5±0.4	South	7	2 198	11 425 585	7 983 164	1 229 183	3,777	116	6	2 414	15 307 536	10 576 813	1 980 462	3,496	101
30.01.2017	08:20-16:10	-6.0±2.3	88±4	0.05	1.5±0.5	North-West	23	9 260	10 155 534	6 717 164	1 036 618	4,438	198	28	12 085	14 421 435	9 462 981	1 457 029	4,477	201
31.01.2017	08:20-16:20	-1.4±0.7	92±2	0	1.4±0.4	North	27	10 986	11 277 740	7 260 346	1 513 678	4,397	224	28	13 125	14 181 509	9 516 845	2 121 891	4,320	215
06.02.2017	08:00-17:00	-0.5±0.6	79±6	0	4.1±1.2	East	16	6 513	15 566 927	10 273 901	2 197 900	4,249	195	22	10 013	24 426 205	16 342 682	3 530 854	4,219	197
10.02.2017	09:00-15:15	-4.3±0.4	86±3	0.1	3.8±0.4	North-East	18	4 853	19 227 002	12 380 516	2 238 452	4,383	183	27	7 233	19 837 152	13 034 122	2 551 770	4,432	194
22.05.2017	08:45-16:45	15.3±1.5	49±7	0	2.4±1.0	South-West	103	24 390	12 607 714	8 354 842	945 334	4,234	118	122	27 270	11 059 366	7 402 821	864 601	4,209	123
23.05.2017	08:25-16:25	15.6±2.6	51±8	0	2.4±1.1	East	112	19 213	9 136 784	5 909 297	474 685	3,902	93	104	20 015	9 579 232	6 457 475	519 945	3,964	99
24.05.2017	08:40-16:40	18.6±2.9	43±9	0	2.4±1.0	South	97	17 548	12 088 280	8 036 780	714 870	4,009	88	120	25 557	11 057 410	7 729 469	699 871	4,028	87

Table S3. Metadata subway air sampling campaign. Eight paired air samples were collected side-by-side using SASS 3100 and ACD-200 Bobcat in the Oslo subway system on 21st June 2017. Each sample was collected for 30 minutes with the air samplers mounted on tripods with an inlet height of ~1.5 meters. SASS and Bobcat were operated at a sampling flowrate of 300 and 200 liters of air per minute (LPM), respectively.

**Location**	**Time of day**	**Latitude**	**Longitude**	**Meteorological conditions**		**SASS 3100**						**ACD-200 Bobcat**					
						**Yield**	**Shotgun metagenomic sequencing (read statistics)**			**Alpha Diversity (species)**		**Yield**	**Shotgun metagenomic sequencing (read statistics)**			**Alpha Diversity (species)**	
				**Temp (°C)**	**RH (%)**	**Total DNA (pg m^{-3})**	**Raw reads (150 bp, paired)**	**Quality filtered reads (≥Q20, ≥100 bp, PhiX and human removed, paired)**	**Classified reads (KrakenUniq, NCBI RefSeq database, paired)**	**Shannon Index**	**Observed Richness**	**Total DNA (pg m^{-3})**	**Raw reads (150 bp, paired)**	**Quality filtered reads (≥Q20, ≥100 bp, PhiX and human removed, paired)**	**Classified reads (KrakenUniq, NCBI RefSeq database, paired)**	**Shannon Index**	**Observed Richness**
Ellingsrudåsen	09:00-09:30	59,93651	10,91664	19.0±0.2	60±1	182	4 060 760	2 993 878	790 624	4,653	312	432	4 167 195	2 394 912	496 220	4,481	289
Lindeberg	09:40-10:10	59,93276	10,88167	15.3±0.3	54±8	274	3 549 641	1 256 016	267 985	4,597	289	592	4 223 095	2 391 942	599 788	3,577	227
Helsfyr	10:27-10:57	59,91259	10,80139	19.4±0.7	41±4	296	4 040 722	2 903 645	721 566	4,842	335	532	4 691 367	2 556 796	553 935	4,789	339
Tøyen	11:07-11:37	59,91558	10,7758	20.6±0.4	54±2	279	4 035 098	2 932 510	938 119	4,281	373	632	4 552 661	2 457 036	595 458	4,390	326
Majorstua	11:53-12:23	59,93016	10,71473	19.1±0.6	35±1	391	3 427 688	2 651 652	611 492	5,022	275	618	4 246 963	2 624 188	549 341	4,792	249
Forskningsparken	12:35-13:05	59,9437	10,72088	20.0±0.7	36±1	222	3 404 937	2 722 437	570 677	4,907	228	292	3 898 530	2 682 657	381 159	4,649	192
Nydalen	13:18-13:48	59,9494	10,76592	19.1±0.3	39±2	309	3 866 835	2 856 383	703 427	4,996	316	573	3 564 427	2 026 702	374 922	4,846	272
Løren	14:10-14:40	59,9301	10,78959	16.3±0.3	57±2	89	3 670 661	2 373 638	580 728	4,589	319	192	3 691 910	2 002 173	406 300	4,677	306
